# Effects of *ABCB1*, *UGT1A1*, and *UGT1A9* Genetic Polymorphisms on the Pharmacokinetics of Sitafloxacin Granules in Healthy Subjects

**DOI:** 10.1002/cpdd.848

**Published:** 2020-07-20

**Authors:** Lu‐Ning Sun, Guo‐Xian Sun, Yu‐Qing Yang, Ye Shen, Feng‐Ru Huang, Li‐Jun Xie, Juan Cheng, Hong‐Wen Zhang, Xue‐Hui Zhang, Yun Liu, Yong‐Qing Wang

**Affiliations:** ^1^ Research Division of Clinical Pharmacology First Affiliated Hospital of Nanjing Medical University Nanjing China; ^2^ Department of Pharmacy Affiliated Hospital of Yangzhou University Yangzhou China; ^3^ Department of Pharmacy Jiangsu Shengze Hospital Suzhou China

**Keywords:** *ABCB1*, gene polymorphism, pharmacokinetics, safety, sitafloxacin, *UGT1A1*, *UGT1A9*

## Abstract

Sitafloxacin, a new fluoroquinolone, has strong antibacterial activity. We evaluated the effects of sitafloxacin granules in single‐dose and multidose cohorts and the effects of *ABCB1, UGT1A1*, and *UGT1A9* genetic polymorphisms on the pharmacokinetics (PK) of sitafloxacin in healthy subjects. The single‐dose study included 3 fasted cohorts receiving 50, 100, and 200 mg of sitafloxacin granules and 1 cohort receiving 50 mg of sitafloxacin granules with a high‐fat meal. The multidose study included 1 cohort receiving 100 mg of sitafloxacin granules once daily for 5 days. PK parameters were calculated using noncompartmental parameters based on concentration‐time data. The genotypes for *ABCB1*, *UGT1A1*, and *UGT1A9* single‐nucleotide polymorphisms were determined using Sanger sequencing. Subsequently, the association between sitafloxacin PK parameters and target single‐nucleotide polymorphisms was analyzed. Sitafloxacin granules were well tolerated up to 200 and 100 mg in the single‐dose and multidose studies, respectively. Sitafloxacin AUC and C_max_ increased linearly within the detection range, and a steady state was reached within 3 days after the administration of multiple oral doses. Our findings showed that C_max_ was lower in the *ABCB1* (rs1045642) mutation group, whereas t_1/2_ was longer in the *UGT1A1* (rs2741049) and *UGT1A9* (rs3832043) mutation groups. In conclusion, sitafloxacin granules were safe at single doses and multiple doses up to 200 and 100 mg/day, respectively, with a linear plasma PK profile. However, *ABCB1* (rs1045642), *UGT1A1* (rs2741049), and *UGT1A9* (rs3832043) genetic polymorphisms are likely to influence the C_max_ or t_1/2_ and thereby merit further clinical evaluation.

Sitafloxacin (DU‐6859a) is a fourth‐generation fluoroquinolone with broad‐spectrum antibacterial activity.^1^, ^2^, ^3^ Sitafloxacin plays a significant role in the management of quinolone‐resistant *Pneumococcus* spp., *Staphylococcus aureus*, *Pseudomonas* spp*.*, and vancomycin‐resistant enterococci.^4^, ^5^, ^6^ Sitafloxacin was first marketed in Japan for the treatment of bacterial infections, including complicated intra‐abdominal abscesses, bronchopneumonia, community‐acquired pneumonia, and skin infections.

Appropriate bioavailability can be achieved with oral doses of sitafloxacin, owing to its solubility characteristics as an amphoteric electrolyte, which enables its rapid absorption.^6^ Several studies have previously demonstrated that sitafloxacin is rapidly absorbed after oral administration and has high bioavailability (89%).^7^, ^8^, ^9^ The results of a sitafloxacin pharmacokinetic (PK) study in healthy volunteers showed that the cumulative urinary excretion of unchanged sitafloxacin within 48 hours after administration was about 70%.^10^ The main metabolite of sitafloxacin is glucuronic acid conjugate, with concentrations of about 30%‐38% in the serum and 5%‐12% in the urine of rats.^6^, ^11^ In vitro study has shown that sitafloxacin has a moderate inhibitory effect on CYP1A but no effects on other CYP450s.^12^ In addition, in vitro studies have shown that UGT1A1 and UGT1A9 enzymes are involved in the glucuronidation of sitafloxacin in human liver microsomes.^13^ Alvarez et al^14^ reported that the drug efflux transporter ATP‐binding cassette B1 (ABCB1) significantly affected the PK disposition of quinolones. Sitafloxacin is a substrate for ABCB1 and can be effluxed after glucuronidation.^15^ However, the influence of genetic polymorphisms in *ABCB1*, *UGT1A1*, and *UGT1A9* on the PK of sitafloxacin has remained unknown.

In this study, we aimed to describe the PK properties as well as safety profiles of the oral doses of sitafloxacin granules and to determine the effects of genetic polymorphisms in *ABCB1*, *UGT1A1*, and *UGT1A9* on the PK characteristics of sitafloxacin following the administration of a single oral dose or multiple oral doses in healthy Chinese subjects.

## Subjects and Methods

### Subjects

A total of 30 subjects were enrolled in the study. All the participants in the study were healthy adult Chinese male and female volunteers aged 18 to 45 years.

#### Principal Inclusion Criteria

The principal inclusion criteria in the study were body weight ≥ 50 kg and body mass index (BMI) ≥ 19 and < 25.0 kg/m^2^ at screening. In addition, all participants were judged by the researcher to be healthy, based on physical examination findings (subjective symptoms and objective findings), body temperature, pulse rate, blood pressure, electrocardiogram, and laboratory tests (clinical chemistry, endocrinology, hematology, and urinalysis) at screening and before administration of the study drug. The subjects fully understood the purpose of the trials, the pharmacological effects of the drug, and the adverse drug reactions, and they voluntarily signed the informed consent.

#### Principal Exclusion Criteria

The study excluded participants with any prior serious cardiovascular, gastrointestinal, renal, or hepatic diseases that could impact the absorption or disposition of sitafloxacin; any prior nervous system disease; any abnormal clinical laboratory results before the start of study; any prior positive screening test results for medicine, tobacco, or alcohol; or a known allergy to any drug, especially to fluoroquinolones. In addition, subjects who were on any prescription or over‐the‐counter medication during the 2 weeks before the trial start date, who had been on any investigational drug in the preceding 3 months, or who were classified as unqualified candidates for the trial by the investigator for any reason before or during the study were excluded. Female subjects who were menstruating or had been confirmed to be pregnant were also ineligible.

### Safety Monitoring

In this study, all subjects were continuously observed at the unit, and details of adverse effects were obtained and recorded by the research personnel. Routine safety assessments were performed at every scheduled study visit to identify adverse effects as indicated by the results of physical examinations, laboratory tests, and electrocardiogram (ECG) measurements. The adverse effects observed during the study could be classified as serious (requiring hospitalization, incapacitating or life‐threatening, or led to death), moderate (uncomfortable feelings that interfered with daily activities), or mild (the participant was aware of a sign or symptom, but it was tolerable). Adverse effects were recorded and managed in accordance with good clinical practice.

### Study Design

This was a single‐center, open‐label, randomized trial carried out from January 2015 to April 2016. The study was conducted at the clinical pharmacology laboratory of the First Affiliated Hospital of Nanjing Medical University, China. The study protocol was approved by the Institutional Review Board of the First Affiliated Hospital of Nanjing Medical University, and the trial identifier was 2014‐MD‐139. The drug clinical trial batch number (2014L00334) was awarded by the National Medical Products Administration. The study was conducted in accordance with the Declaration of Helsinki and Good Clinical Practices consolidated guidelines. All subjects provided written informed consent before participating in any study procedures. Subjects could withdraw from the study at their own request for safety, for behavioral or administrative reasons, or for any other reason at the discretion of the investigator or sponsor.

#### Single‐Dose Study

The safety of sitafloxacin was assessed by an open‐label, single‐dose clinical trial (Table 1). The study population was 50% male and 50% female and was randomly divided into 50‐, 100‐, and 200‐mg dose groups with 10 subjects in each group. An initial dose of 50 mg was orally administered to the subjects, which was increased when the 50‐mg dose was verified to be safe.

**Table 1:** Demographic Characteristics and Genotype Study Group

Demographic Characteristics	Single Dose, 50 mg	Single and Multiple Doses, 100 mg	Single Dose, 200 mg	Summary
Age,^a^ year	23.9 (3.5), [18‐30]	24.7 (3.5), [19‐29]	25.1 (3.4), [20‐30]	24.7 (3.4), [18‐30]
Weight,^a^ kg	58.0 (5.5), [52‐67]	59.8 (5.5), [54‐72]	59.2 (6.3), [50.5‐69]	59.0 (5.7), [50.5‐72]
Height,^a^ m	1.66 (0.06), [1.58‐1.77]	1.66 (0.06), [1.58‐1.75]	1.67 (0.06), [1.58‐1.74]	1.66 (0.06), [1.58‐1.75]
BMI,^a^ kg/m^2^	21.1 (1.4), [19.2‐23.4]	21.7 (1.2), [19.6‐24.3]	21.2 (1.6), [19.2‐23.3]	21.3 (1.4), [19.2‐24.3]
Sex, n (%)				
Male	5 (50)	5 (50)	5 (50)	15 (50)
Female	5 (50)	5 (50)	5 (50)	15 (50)
Race, n (%)				
Han	10 (100)	10 (100)	10 (100)	30 (100)
*ABCB1*				
rs10248420	GG (2)	GG (2)	GG (1)	GG (5)
	AA (3)	AA (3)	AA (3)	AA (9)
	GA (5)	GA (5)	GA (6)	GA (16)
*ABCB1*				
rs1045642	CC (3)	CC (5)	CC (3)	CC (11)
	TT (3)	TT (1)	TT (1)	TT (5)
	TC (4)	TC (4)	TC (6)	TC (14)
*ABCB1*				
rs1128503	CC (2)	CC (1)	CC (1)	CC (4)
	TT (6)	TT (6)	TT (4)	TT (16)
	TC (2)	TC (3)	TC (5)	TC (10)
*ABCB1*				
rs2032582	GG (2)	GG (4)	GG (1)	GG (7)
	G/A·T (3)	G/A·T (3)	G/A·T (7)	G/A·T (13)
	T·A/ T·A (5)	T·A/ T·A (3)	T·A/ T·A (2)	T·A/ T·A (10)
*UGT1A1*				
rs8175347	AA (10)	AA (10)	AA (9)	AA (29)
	TA (0)	TA (0)	TA (1)	TA (1)
*UGT1A1*				
rs887829	GG (9)	GG (10)	GG (8)	GG (27)
	GA (1)	GA (0)	GA (2)	GA (3)
*UGT1A9*				
rs2070959	AA (5)	AA (5)	AA (6)	AA (16)
	GG (1)	GG (0)	GG (0)	GG (1)
	GA (4)	GA (5)	GA (4)	GA (13)
*UGT1A9*				
rs2741049	TT (4)	TT (4)	TT (4)	TT (12)
	CC (3)	CC (2)	CC (2)	CC (7)
	TC (3)	TC (4)	TC (4)	TC (11)
*UGT1A9*				
rs3806598	TT (6)	TT (5)	TT (6)	TT (17)
	GG (1)	GG (0)	GG (0)	GG (1)
	GT (3)	GT (5)	GT (4)	GT (12)
*UGT1A9*				
rs3832043	TT (5)	TT (1)	TT (3)	TT (9)
	AA (5)	AA (9)	AA (7)	AA (21)
*UGT1A9*				
rs6759892	GG (1)	GG (0)	GG (0)	GG (1)
	TT (5)	TT (5)	TT (5)	TT (15)
	GT (4)	GT (5)	GT (5)	GT (14)

BMI, body mass index.

a Expressed as mean (SD), [range].

#### Multiple‐Dose Study

An open‐label clinical trial was performed following the 100‐mg single‐dose group. The same 10 subjects remained in the multidose study. A single dose of the drug was administered on the first day, and no dose was administered on the second day. From the third day of the trial, the subjects were administered 100 mg once a day for 5 consecutive days.

#### Food Effect Study

A single‐dose (50‐mg), open‐label, randomized, 2‐period, 2‐sequence crossover study was designed to investigate the effects of nutrition (fasting or a high‐fat meal). During the first period, 5 randomly chosen subjects were given a high‐fat meal for breakfast within a half‐hour before drug administration, and they were compared with other fasting subjects. During the second period, subjects who were fasted during the first period were given a high‐fat meal, whereas the other subjects were fasting. This study also included a washout period of 5 days to separate the 2 periods.

### Sample Collection and Analysis

For the single‐dose groups (50, 100, and 200 mg), blood samples were taken before dosing and 10, 20, 30, and 45 minutes and 1, 1.25, 1.5, 2, 3, 5, 8, 12, 24, and 36 hours after the initial dose. For the multiple‐dose group (100 mg), blood samples were collected before dosing on days 5 through 6, and on days 1 and 7, blood samples were collected at the same times as in the single‐dose group. Drug concentration was not measured in the single‐dose group before the administration of drug to the multiple‐dose group. Plasma was also isolated by centrifuging the blood samples at 3500*g* for 8 minutes at 4°C and was stored at approximately −80°C. For the 50‐mg single‐dose group, urine samples were collected at predose (0 hours) and 0‐3, 3‐6, 6‐12, 12‐24, 24‐36, 36‐48, and 48‐60 hours after oral dosing. Urine volume was recorded, and the samples were stored at approximately −80°C until analysis. If the time of blood collection overlapped with the time of urine collection, priority was given to blood collection. All the experimental procedures were carried out in the dark.

### Analytical Assay

Plasma and urine samples were analyzed with validated high‐performance liquid chromatography‐tandem mass spectrometry (HPLC‐MS/MS) methods using Applied Biosystems/Sciex Triple Quad 5500 (Foster City, California). The compounds were separated on a Poroshell 120 SB‐C18 column (50 × 2.1 mm, 2.7 μm; Agilent, Santa Clara, California) at 38°C. The internal standard was moxifloxacin (purity, 96.1%; European Pharmacopeia reference standard), which was dissolved in methanol‐water (50:50) and diluted with formic acid (chromatographic purity, Darmstadt, Germany, Merck Company) and methanol (chromatographic purity; Los Angeles, California, Aladdin Company). The mobile phase consisted of water containing 0.1% formic acid and methanol (62:38, v/v). For the analysis, 1.0 μL of sample was injected into the HPLC‐MS/MS system using electrospray ionization in positive mode. The multiple reaction monitoring transitions for sitafloxacin and internal standard (moxifloxacin) were 410.1→392.0 m/z and 402.2→384.1 m/z, respectively. The total run time per sample was 2.5 minutes at a flow rate of 0.5 mL/min. Data processing, statistics, and calculations were performed using Analyst 1.6.2. software (Sciex, Redwood, California). The standard curves of sitafloxacin were linear over the concentration ranges of 10.03‐4010 ng/mL and −0.2503 to 200.2 μg/mL in the plasma and urine samples, respectively. The intra‐ and interbatch precision and accuracy values (<15%) met the acceptance criteria according to the Guidance for Industry, Analytical Procedures and Methods Validation for Drugs and Biologics.^16^

### PK Analysis

The calculation of PK parameters was performed with DAS software (version 2.1.1 Hefei, China) using noncompartmental analysis techniques. The following PK parameters were calculated: area under the concentration‐time curve (AUC) from 0 to t (AUC_0‐t_), AUC from time 0 extrapolated to infinity (AUC_0‐∞_), steady‐state AUC (AUC_(0‐tau)ss_), maximum observed concentration (C_max_), time to C_max_ (T_max_), the minimum value of the steady plasma drug concentration (C_min_), elimination half‐life (t_1/2_), the average steady‐state concentration (AUC), the degree of fluctuation (DF; [C_ss max_ − C_ss min_]/C_av_). For the PK analysis, the prepeak below low quantitation (BLQ) samples were assigned values of zero, and the postpeak BLQ samples were processed as no data. When drawing the individual concentration‐time curves, the BLQ samples were all treated as no data. In the statistical analysis, the BLQ samples were all assigned values of zero.

### Genotyping

A total of 11 single‐nucleotide polymorphisms (SNPs), 4 for ABCB1, 2 for UGT1A1, and 5 for UGT1A9, were selected based on previous evidence of functional significance on drug response using the relevant literature and PharmGKB database.^17^, ^18^, ^19^ Whole‐blood samples were collected from 30 patients to obtain genomic DNA. Genomic DNA was extracted using a Tiangen (Beijing, China) genomic DNA purification kit. Next, we genotyped SNPs in the following genes: *ABCB1*, rs1128503, rs10248420, rs2032582, rs1045642; *UGT1A1*, rs887829 and rs8175347; and *UGT1A9*, rs2070959, rs2741049, rs3806598, rs3832043, and rs6759892. SNP coordinates were obtained from the National Biotechnology Information Center SNP database (https://www.ncbi.nlm.nih.gov/projects/SNP/). The SNPs were profiled using Sanger sequencing technology at the Center for Shanghai Sangon Bio‐Tech Co., Ltd. (https://www.sangon.com/ [in Chinese]). Detailed information about the genotyping protocol can be found in a previous study.^20^ All the subjects’ SNPs were successfully genotyped with a call rate of 100%.

### Statistical Analysis

All analyses were carried out using SPSS version 11.0 (SPSS, Inc., Chicago, Illinois). PK parameters, including AUC and C_max_, were calculated using the natural logarithms of individual values before the analysis. To explore dose proportionality, analysis of covariance was used to determine the 90% confidence intervals (CIs), and the slope β was provided by the power model: ln(PK) = β_0_ + β_1_ × ln(dose). A regression coefficient at a level of 0.1 was considered significant. This study used a predefined standard of (0.5, 2.0)^21^, ^22^ and a standard interval of (0.500, 1.500). Across the different dose groups, the relevant PK parameters were calculated using an analysis of variance (ANOVA) except for T_max_, which was calculated using a nonparametric test. A paired *t* test was used to compare the PK parameters between single and multiple doses, and an independent *t* test was used to compare the PK parameters between the male and female subjects. To determine the steady state in the multiple‐dose group, ANOVA was used to compare the differences in C_min‐ss_ on days 5, 6, and 7. The PK parameters of each dose group were divided into different groups according to sex, and an independent‐sample *t* test (T_max_ nonparametric test) was performed. The PK parameters of the male and female subjects in each dose group were then statistically analyzed.

The Kolmogorov‐Smirnov and Levene tests were used to evaluate the distribution of continuous data and the homogeneity of variance, respectively, with parametric and nonparametric tests applied appropriately. Comparisons of the PK parameters among the groups that were genotyped for *ABCB1*, *UGT1A1*, and *UGT1A9* were performed using 1‐way ANOVA and nonparametric Mann‐Whitney, and Kruskal‐Wallis tests. A post hoc Duncan test or a Dunn test was used to determine if there was a significant difference between each of the genotypic groups when a statistically significant association was identified by a Kruskal‐Wallis or ANOVA test, respectively. A *P* < .05 indicated statistical significance.

## Results

### Study Population

The demographic features of the subjects are presented in Table 1. Thirty healthy subjects were involved in the study; none of them withdrew.

### Safety

Sitafloxacin was safe across the 3 dose groups (50, 100, and 200 mg). Drug‐related adverse events, including hypersensitivity, gastrointestinal tract reaction, phototoxicity, and central nervous system effect, were not observed during the study. There were no significant changes in the mean baseline values of the clinical laboratory variables (clinical chemistry, coagulation, hematology, stool routine, and urinalysis) or vital signs. The values outside the normal ranges were not clinically meaningful. In addition, there were no clinically significant changes in the heartrate or ECG measurements during the study.

### Single‐Dose Study

**Figure 1:**
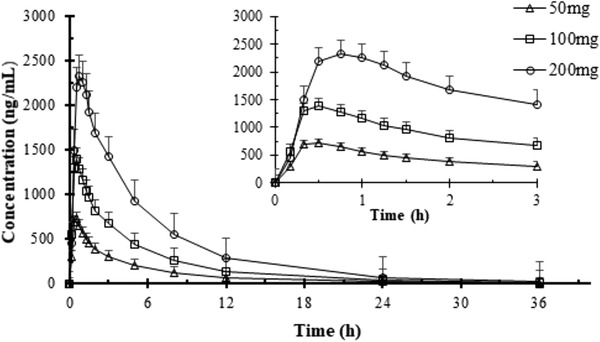
Mean plasma concentration‐time curve of single dose of sitafloxacin (50, 100, 200 mg) mean ± SD. Plasma concentrations below the limits of quantitation were treated as zero.

The mean plasma drug concentration‐time curve is presented in Figure 1. The mean (standard deviation [SD]) values of PK parameters are shown in Table 2. Sitafloxacin was rapidly absorbed, with an average T_max_ of 0.48‐0.83 hours across the different dose groups. The mean t_1/2_ at doses ≥ 100 mg ranged from 4.94 to 5.58 hours, whereas that at the lowest dose (50 mg) was 4.36 hours. The C_max_ values of the 50‐ and 200‐mg doses of sitafloxacin were 757.9 and 2612 ng/mL, respectively, and their corresponding AUC_0‐∞_ values were 3330 and 14 169 ng·h/mL, respectively. In the 50‐mg dose group, 70.0% ± 6.4% sitafloxacin was recovered from the urine within 60 hours after drug administration (Table 2).

**Table 2:** In Vivo Pharmacokinetic Parameters of Sitafloxacin After Single and Multiple Doses of Oral Sitafloxacin

	50 mg (n = 10)						100 mg (n = 10)						200 mg (n = 10)		
	Fasting, Single Dose			Fed, Single Dose			Fasting, Single Dose			Fasting, Multiple Dose			Fasting, Single Dose		
Parameters	Male	Female	All	Male	Female	All	Male	Female	All	Male	Female	All	Male	Female	All
C_max_ (ng/mL), mean (SD)	702.9 (121.8)	812.9 (127.1)	757.9 (130.9)	359.4 (49.9)	374.2 (20.2)	366.8 (36.7)	1492 (352)	1476 (141)	1484 (253)	1213 (409)	1265 (237)	1239 (317)	2194 (298)	3029 (328)	2612 (530)
T_max_ (h), mean (SD)	0.53 (0.21)	0.43 (0.09)	0.48 (0.16)	1.95 (0.8)	2.80 (0.45)	2.38 (0.76)	0.47 (0.08)	0.55 (0.31)	0.51 (0.22)	0.67 (0.36)	0.57 (0.25)	0.62 (0.30)	0.80 (0.33)	0.85 (0.34)	0.83 (0.31)
t_1/2_ (h), mean (SD)	4.74 (1.36)	3.99 (0.42)	4.36 (1.03)	5.87 (1.11)	3.61 (0.14)	4.74 (1.40)	5.52 (1.44)	4.36 (1.35)	4.94 (1.45)	7.04 (1.85)	4.88 (1.53)	5.96 (1.96)	6.18 (0.55)	4.97 (0.22)	5.58 (0.75)
AUC_0‐t_ (ng·h/mL), mean (SD)	3325 (1311)	3179 (444)	3252 (926)	3050 (946)	2752 (213)	2901 (665)	7139 (1758)	6815 (776)	6977 (1293)	7517 (1992)	6657 (536)	7087 (1448)	12 591 (1175)	15 481 (2692)	14 036 (2481)
AUC_0‐∞_ (ng·h/mL), mean (SD)	3438 (1321)	3222 (448)	3330 (937)	3191 (971)	2785 (217)	2988 (697)	7269 (1829)	6873 (788)	7071 (1344)	7754 (2176)	6733 (601)	7243 (1598)	12 769 (1178)	15 570 (2691)	14 169 (2453)
Ae (%), mean (SD)	72.7 (2.1)	67.3 (8.3)	70.0 (6.4)	68.5 (5.0)	62.6 (15.6)	65.9 (10.7)	n/a	n/a	n/a	n/a	n/a	n/a	n/a	n/a	n/a
C_min_ (ng/mL), mean (SD)	n/a	n/a	n/a	n/a	n/a	n/a	n/a	n/a	n/a	57.53 (37.42)	33.17 (13.47)	45.35 (29.46)	n/a	n/a	n/a
C_av_ (ng/mL), mean (SD)	n/a	n/a	n/a	n/a	n/a	n/a	n/a	n/a	n/a	296.1 (71.5)	269.6 (18.5)	282.8 (51.2)	n/a	n/a	n/a
AUC_ss_ (ng·h/mL), mean (SD)	n/a	n/a	n/a	n/a	n/a	n/a	n/a	n/a	n/a	7106 (1717)	6469 (444)	6788 (1229)	n/a	n/a	n/a
DF, mean (SD)	n/a	n/a	n/a	n/a	n/a	n/a	n/a	n/a	n/a	3.955 (1.155)	4.614 (1.096)	4.285 (1.117)	n/a	n/a	n/a

AUC, area under the curve; AUC_0‐t_, AUC from 0 to last quantifiable time; AUC_0‐∞_, AUC from 0 to infinity; AUC_ss_, steady‐state AUC over the dosing interval; C_max_, maximum plasma concentration; C_min_, minimum plasma concentration; C_av_, average concentration; Ae, cumulative excretion; t_max_, time to C_max_; t_1/2_, elimination half‐life; DF, degree of fluctuation; DF = (C_ss max_ − C_ss min_)/C_av_.

Across the dose range used in this study, the mean C_max_, AUC_0‐t_, and AUC_0‐∞_ increased linearly with the dose, as shown by the linear regression analysis. A proportional dose‐response relationship was also observed (*P* > .05) by an analysis of variance of ln(C_max_/dose) and ln(AUC/dose) of the 3 dose groups. In addition, the mean slope of ln(dose) versus ln(AUC) or ln(C_max_) was close to 1, and the slopes of the 90%CIs were fully controlled within a predetermined dose ratio range (0.5000‐1.500). The mean slopes (90%CIs) of the parameters were: C_max_, 0.942 (0.842‐1.041); AUC_0‐t_, 1.106 (1.001‐1.210); and AUC_0‐∞_, 1.102 (0.996‐1.207). Therefore, AUC and C_max_ were proportional to the study doses that were tested in disparate ways. As the dose increased, absorption and elimination of sitafloxacin decreased.

### Multiple‐Dose Study

**Figure 2:**
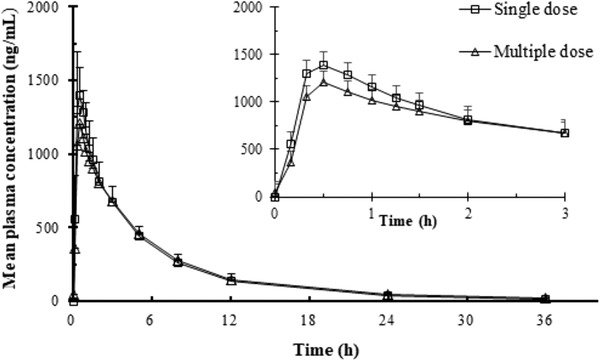
Mean plasma concentration‐time curve of single and multiple doses of sitafloxacin (100 mg) mean ± SD. Plasma concentrations below the limits of quantitation were treated as zero.

The mean drug concentration‐time curve in plasma after the first and last administration of sitafloxacin is shown in Figure 2, and the PK parameters of the noncompartmental analysis of the plasma drug concentrations that were measured on days 1 and 7 are summarized in Table 2.

The C_ss‐min_ was not significantly different according to ANOVA, which indicated that once‐daily administration of 100 mg of sitafloxacin reached a steady state within 3 days. Sitafloxacin was rapidly absorbed under steady‐state conditions, with a mean C_max_ of 1239 ng/mL and a mean T_max_ of 0.62 hours, which were the same as those of single‐dose parameters (day 1). Plasma sitafloxacin was removed in a biphasic manner with no obvious differences in the t_1/2_ or AUC between the first and the last dose. The DF of plasma sitafloxacin was 4.285.

### Effects of Food and Sex

**Figure 3:**
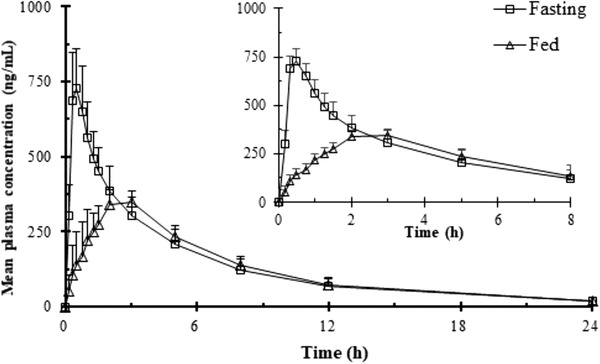
Mean plasma concentration‐time curve of single‐dose pharmacokinetic study (50 mg fasting and fed by a high‐fat meal) mean ± SD. Plasma concentrations below the limits of quantitation were treated as zero.

A 50‐mg dose of sitafloxacin was administered with either high‐fat food or under fasting condition, and the results showed that the high‐fat group experienced about a 50% reduction in the mean ± SD C_max_ from 757.9 ± 130.9 to 366.8 ± 36.7 ng/mL (Figure 3). The absorption (T_max_), elimination (t_1/2_), and total exposure (AUC_0‐t_ and AUC_0‐∞_) were not affected by dosing under the different feeding conditions.

There was a significant difference in t_1/2_ and C_max_ between the male and female subjects only in the 200‐mg dose group (*P* < .05). However, we also observed that sex did not affect AUC_0‐t_, AUC_0–∞_, or T_max_ after a single oral administration of sitafloxacin granules in the 50‐, 100‐, or 200‐mg groups (*P >* .05). As the dose increased, the male group showed a lower C_max_ and a higher t_1/2_ than the female group. For the multiple‐dose group, sex had no significant effects on the C_max_, AUC, t_1/2_, and T_max_ (*P* > .05).

### Genetic Polymorphisms and PK

Thirty healthy Chinese adults were enrolled in the study. There were no significant differences in age or BMI between the genotypes. The genotype groups of the selected genes are shown in Table 1. All the genotypes were in Hardy‐Weinberg equilibrium (*P >* .05).

**Figure 4:**
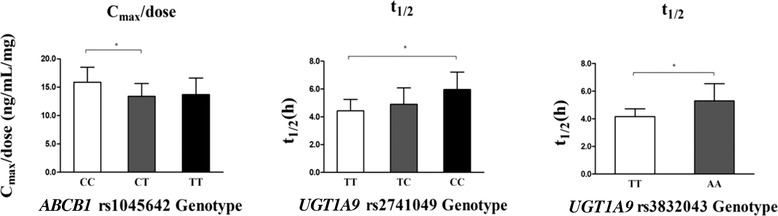
Comparison of the PK parameters of sitafloxacin in different genotype groups.

The effects of genetic polymorphisms in *ABCB1* (rs1045642, rs1128503, rs2032582, rs10248420), *UGT1A1* (rs887829, rs8175347), and *UGT1A9* (rs2070959, rs2741049, rs3806598, rs3832043, rs6759892) on the PK of sitafloxacin were evaluated. *ABCB1* (rs1045642) had a significant effect on the C_max_/dose, and *UGT1A9* (rs2741049, rs3832043) had a significant effect on t_1/2_ (*P* < .05); however, the other PK parameters were not affected (Figure 4).

## Discussion

In this study, sitafloxacin was well tolerated, and repeated use of sitafloxacin did not cause any adverse effects in the healthy subjects.

Sitafloxacin was rapidly absorbed, and the blood concentrations showed a biphasic and downward trend that was consistent with the results of previous studies.^23^ Furthermore, the AUC and C_max_ of sitafloxacin increased proportionally across the range of study doses, whereas the T_max_ and t_1/2_ values remained unaffected.

During the repeated‐dose study, a steady‐state blood concentration was reached within 3 days. The values of PK parameters for single and multiple doses of sitafloxacin were similar. We observed blood concentrations of sitafloxacin with a high DF, and these concentrations rapidly decreased from T_max_ to t. These data were consistent with those of other PK studies of sitafloxacin in Japan and China.^6^, ^10^, ^23^

Administration of fluoroquinolones with food can delay absorption.^24^ The effects of a high‐fat diet on the PK parameters of sitafloxacin were studied using fine granules. The T_max_ of sitafloxacin was prolonged when taken with food, which was likely caused by a delay in gastric emptying. The C_max_ decreased significantly (50%), but no significant changes in the AUC were observed. These results indicate that it is better to administer sitafloxacin under fasting when using the sitafloxacin granules. There were no sex‐influenced differences in the AUC or T_max_ values, but with increased dose, the male group had a lower C_max_ and a higher t_1/2_ compared with the female group. The sex‐influenced differences in some of the PK parameters at high dose were mainly from the degree of elimination; however, there were no significant sex‐influenced differences in the AUC_0‐t_, AUC_0‐∞_, and T_max_ values of sitafloxacin in healthy Chinese subjects. This implies that the degree of elimination of sitafloxacin would differ as the dose increases.

To the best of our knowledge, no previous studies have explored the effects of genetic polymorphisms in ABCB1 transporter or in UGT1A1 and UGT1A9 phase II drug‐biometabolizing enzymes on the PK parameters of sitafloxacin in humans. ABCB1 is an efflux transporter that primarily affects drug clearance.^25^, ^26^ It is highly expressed in the apical hepatocyte membrane, intestinal epithelium, placenta, and several other organs.^27^ Sitafloxacin is a substrate for ABCB1; several studies have reported that ABCB1 is capable of transporting quinolone‐based antibacterial drugs and that it may promote renal tubular secretion to some extent and fulfill other elimination processes.^14^, ^15^ Here, we report a novel association between the genetic polymorphism of the human drug transporter *ABCB1* (rs1045642) and sitafloxacin exposure and/or peak concentration. We showed that the C_max_ of sitafloxacin in subjects who were heterozygous or homozygous for the *ABCB1* (rs1045642) variant was significantly lower than that in subjects without the variant (*P <* .05). Although there was no significant difference between the AUC_0‐∞_/dose of the variant group and the group without the variant, there were significant trends across the 3 groups of parameters, which suggests that the expression and function of *ABCB1* (rs1045642) may be increased in the variant group. This result is consistent with previously reported trends of allelic mutations that caused functional changes.^27^

UDP‐glucuronosyltransferase (UGT) enzymes are important phase II drug‐biometabolizing enzymes. The catalytic substrates of these enzymes bind to uridine diphosphate glucuronic acid groups to increase their hydrophilicity and facilitate their excretion from the body.^28^ The UGT1A family is located on Chr2 (2q37) and has 13 members in humans. All its members contain exon 1, and the sequence determines the function of the protein encoded by the UGT1A family. Previous studies have shown that UGT1A1, UGT1A3, and UGT1A9, particularly UGT1A1, are involved in the glucuronidation of sitafloxacin. In addition, UGT1A3 demonstrates relatively low levels of glucuronidation activity. Northern blot analysis of human tissue samples indicated that the expression of UGT1A1 was 20‐fold greater than that of UGT1A3, which indicates that UGT1A3 plays a minor role in the glucuronidation of sitafloxacin in human liver microsomes.^13^ In the *UGT1A1* (rs2741049) and *UGT1A9* (rs3832043) groups, we found that the subjects had a longer t_1/2_ for sitafloxacin than those without the variants (*P <* .05), which was likely because of the reduction of metabolism and disposition of sitafloxacin by the SNP mutations. The results showed that there was no significant difference in the AUC values of the *ABCB1* and *UGT1A* groups and no significant change in net exposure (total AUC). We believe that the reduction in C_max_ and the delay in T_max_ could not be considered to have practical clinical significance.

A possible limitation of the current study was the limited number of subjects. After stratification, the number of subjects in some genotypic groups was low, which limits the reliability of the analyses and comparisons of the genotypic effects. Moreover, all the subjects were young, and the results of this study cannot be generalized to the elderly or patients with infection. As a result, further studies with larger study populations are necessary.

## Conclusions

In summary, the oral dose of sitafloxacin granules was well tolerated in a population of healthy subjects. Sitafloxacin showed good absorption properties, and our findings support the once‐daily dose. The genotypes *UGT1A* (rs2741049), *UGT1A* (rs3832043), and *ABCB1* (rs1045642) were associated with significant differences in sitafloxacin concentrations and t_1/2_. Sitafloxacin exhibits concentration‐dependent bactericidal activity, and hence, slight changes in drug concentrations are not likely to influence its efficacy.

## Conflicts of Interest

The authors declare no conflicts of interest.

## Supporting information

Additional supplemental information can be found by clicking the Supplements link in the PDF toolbar or the Supplemental Information section at the end o f web‐based version of this article.
