# Supplementary material for: Effects of ABCB1, UGT1A1, and UGT1A9 Genetic Polymorphisms on the Pharmacokinetics of Sitafloxacin Granules in Healthy Subjects
Source: Clin Pharmacol Drug Dev. 2020 Jul 20;10(1):57–67. doi: 10.1002/cpdd.848 (PMC7818398; doi:10.1002/cpdd.848)
Supplement: Supplementary file 1 — Additional supplemental information can be found by clicking the Supplements link in the PDF toolbar or the Supplemental Information section at the end o f web‐based version of this article. [file CPDD-10-57-s001.docx]

| **Primer sequences.** | | |
| --- | --- | --- |
| **Genes** | **Primer ID** | **Primer sequences** |
| ABCB1 | rs10248420-F | CACTCACTTTATTCCAGCCACTCTG |
|  | rs10248420-R | ACAGAAGGGAGGGAAGACAATCCT |
| ABCB1 | rs1045642-F | AAGTGTGCTGGTCCTGAAGTTGA |
|  | rs1045642-R | CAGGTAAGGGTGTGATTTGGTTGCT |
| ABCB1 | rs1128503-F | CGAAGAGTGGGCACAAACCAGA |
|  | rs1128503-R | GACTGCTGATCACCGCAGGGT |
| ABCB1 | rs2032582-F | TGCAGGCTATAGGTTCCAGGCT |
|  | rs2032582-R | AGAGCATAGTAAGCAGTAGGGAGT |
| UGT1A1 | rs8175347-F | CCCCACACTGTGCTGGACTCAA |
|  | rs8175347-R | CCCAAGCATGCTCAGCCAGTG |
| UGT1A1 | rs887829-F | GTCAGTCCACAAAGGTAGCAGGGA |
|  | rs887829-R | TCACCTCATGGCGCGTGCT |
| UGT1A9 | rs2070959-F | GCTCCGAGCCGGGTATCACTTC |
|  | rs2070959-R | ACCCAGCCTTACCCTGTGGG |
| UGT1A9 | rs2741049-F | GTGCCAATGCGTGTACTCGTC |
|  | rs2741049-R | CACGGCCCTATTCATAAAGACAA |
| UGT1A9 | rs3806598-F | TGGTCTTTGCCTTGGGGGCA |
|  | rs3806598-R | GGCACTGGAGTGATGGCGTG |
| UGT1A9 | rs3832043-F | GCATTGCAGAGACACAGGCGAG |
|  | rs3832043-R | CTGCCTCGGCAAAGCCACAG |
| UGT1A9 | rs6759892-F | CGGGATAGAGAATTGGCAGGGGG |
|  | rs6759892-R | GACCCCGGTCACTGAGAACCTC |
| F: Forward; R: Reverse | | |
